# Benjamin Rush

**Published:** 1904-08

**Authors:** 


### Benjamin Rush.

AFTER many years of hard work on the part of several prominent members of the American medical profession a monument to one of the earliest physicians of distinction, Dr. Benjamin Rush, has been erected in the grounds of the United States naval museum of hygiene and the medical school. The statue is the gift of the American Medical Association and was unveiled at 5 o'clock p. m., Saturday, June 11, 1904, in the presence of about 500 persons, including the President of the United States, members of the cabinet, officers of the army and navy, and members of the American Medical Association.

Dr. J. H. Musser, president of the American Medical Association, opened the exercises in a brief address at the end of which he introduced Dr. J. C. Wilson, of Philadelphia, who delivered an oration on the life and character of Benjamin Rush. At the conclusion of this address Mr. Louis R. Metcalf, the architect of the statue, unveiled the work while the marine band played "My Country 'tis of Thee."

President Roosevelt was then introduced by Dr. Musser and accepted the statue in the following language:

I accept on behalf of the nation the gift so fittingly bestowed by one of the great professions—this statue of a man who was eminent not only in that profession, but eminent in his service to the nation as a whole. We have listened to the interesting study of the life of Benjamin Rush, and it must surely have been brought home to each of us here that his career derives its peculiar significance in part from the greatness of his pioneer work as a physician on this continent, in part from the way in which he combined with arduous and incessant labor in his profession the greatest devotion even outside of that profession to the welfare of his fellow countrymen.

Here, at the national capital, it is earnestly to be hoped that we shall finally see commemorated, as the services of Rush are henceforth to be commemorated by this statue, all the great Americans, who, working in widely different lines, by the aggregate of their work, make the sum of achievement of America in the world. I thank and congratulate you of the medical profession today upon what you have done, not merely in commemorating the foremost pioneer in your own profession, but in adding at the national capital a figure to the gallery of great Americans who should be here commemorated. (Applause.)

As you said, Dr. Benjamin Rush was not a specialist in the modern sense. He could not be. There were not any specialists in the modern sense, as you pointed out. There was no possibility of there being such. But I would like, in this age of specialisation, to say one word in the way of a short sermon to eminent specialists. Today, no specialist in a democratic country like ours can afford to be so exclusively a specialist as to forget that one part of his duty is his duty to the general public and to the state.

Where government is the duty of all, it of course means that it is the duty of each, and, the minute that the average man gets to thinking that government is the duty of somebody else that minute the republic will begin to go down. It is a fortunate thing for our country that we should have before us the lives of men like Rush, who could take a part in our public life as distinguished as is implied by having been a signer of the Declaration of Independence, and yet do it without a particular neglect of the man's own proper duties. (Applause.)

I would earnestly plead, in addressing this audience, and especially the members of the high and honorable profession which has given this gift to the nation, that you never for one moment permit yourselves to forget the fact that the well being of the republic ultimately depends upon the way in which, as a rule and habitually, the best citizen of the republic does his duty to the state (applause) ; and that we have a right not merely to expect but to demand from our hardest worked men, from the leaders of the great professions, the full performance of that public service which consists in a zealous, intelligent and fearless performance of the ordinary duties of public life by the ordinary private citizen. (Applause.)

I thank you for having presented to the national capital, to the people of the United States, the statue of a man who was foremost as a leader and a pioneer in his profession, who was a great physician and a great American.

This brought to end one of the most interesting ceremonies that has fallen to the lot of the American medical profession to participate in. It witnessed the culmination of the hopes, ambitions, and energies of a group of earnest physicians who have struggled against odds for twenty years to bring about the result thus happily accomplished.

Many of the original projectors of and workers for this consummation have gone to their reward. To those familiar with the inception and early history of the Rush monument scheme, the thought will instinctively arise "Oh, that Gihon, the erudite and genial surgeon who served his country so long and faithfully in the navy, could have lived to witness the scene above described!" For it was to the indefatigable energy, even persistency, of Commodore Albert L. Gihon, medical director, United States Navy, who served for more than fifteen years as chairman of the Rush monument committee, that the work has been brought to such a happy conclusion. He was ably seconded by George H. Rohé, and other members of the committee, but almost everyone knows that Gihon, though met by discouragements and even ridicule, laid the foundation so deep and firm for the building of the statue that those who have finished his work have done so with comparative ease. We should not enjoy the splendid art tribute to the memory of our distinguished colonial physician unless, in this record of its unveiling, we recalled the work in relation to its building of ALBERT LEARY GIHON.

